# The Availability and Affordability of Cardiovascular Medicines for Secondary Prevention in Tehran Province (Iran)

**Published:** 2018

**Authors:** Ali Vasheghani Farahani, Jamshid Salamzadeh, Hamid Reza Rasekh, Sheyda Najafi, Vahideh Mosadegh

**Affiliations:** a *Department of Pharmacoeconomics and Pharmaceutical Management, School of Pharmacy, Shahid Beheshti University of Medical Sciences.*; b *Department of Clinical Pharmacy, School of Pharmacy, Shahid Beheshti University of Medical Sciences. *; c *Department of Pharmaceutical Care, Imam Khomeini Hospital Complex, Tehran University of Medical Sciences, Tehran, Iran. *; d *Islamic Azad University, Ahar Branch, Ahar, Iran.*

**Keywords:** Availability, Affordability, Cardiovascular Diseases, Secondary Prevention

## Abstract

Availability and affordability of medicines are crucial to achieving success in prevention programs, particularly in developing countries. The aim of this study was to determine the availability and affordability of cardiovascular medicines for secondary prevention in Tehran province of Iran. A cross-sectional survey was conducted in Tehran province in 2015, using the 2nd edition of the World Health Organization/Health Action International methodology. Data on the availability and affordability of 21 selected cardiovascular medicines were collected from the public and private healthcare sectors. A total of 120 facilities were included in the survey and the medicines in this survey were both original and lowest-price generic. Lowest-price generic equivalent medicines were highly available (> 80%) in almost all pharmacies of both public and private sectors, while the availability of original brand medicines was highly poor in public and private pharmacies. The median price ratios were 0.72 to 0.76 for generic medicines. The treatment of cardiovascular diseases with lowest-price generic equivalent medicines was generally affordable; moreover, less than a single day’s wage was adequate to purchase a monthꞌs supply of the lowest priced generic of the surveyed medicines. The availability of the selected generic medicines for the secondary prevention of cardiovascular diseases is high in both public and private sectors and they were affordable for low-paid unskilled government workers in the province. The result of this study demonstrates that the supply policies pertaining to generic medicines have been implemented successfully.

## Introduction

Cardiovascular disease (CVD) is among the major causes of mortality around the world. The incidence of CVD is anticipated to increase to approximately 25 million by 2030 (1, 2). The burden of CVD has been mostly shouldered by low- and middle-income countries (LMICs), like Iran, making up for approximately 80% of the annual cardiovascular deaths (3). 

The Middle East and certain parts of Eastern Europe have the highest rates of cardiovascular deaths over the world (4). Ischemic heart disease is the leading cause of mortality in the Middle East, particularly Iran (5). The high prevalence of CVD in LMICs largely reflects the significance of key risk factors, including hypertension, tobacco, unhealthy diet, obesity, and physical inactivity (3). 

Many of these deaths could be prevented through secondary prevention with proven medicines (6). Based on major practice guidelines from the National Institutes of Health and updated ACCF/AHA practice guidelines, and results from recent clinical trials, AHA/ACCF guideline recommends four medicines for the secondary prevention of cardiovascular disease including Aspirin, β blockers, Angiotensin-converting enzyme (ACE) inhibitors or Angiotensin-II receptor blockers (ARBs), and Statins. Several studies have confirmed the benefits of these medicines in the reduction of CVD-induced mortality and morbidity (6-8). 

The World Health Organization (WHO) PREMISE which studied the secondary prevention of CVD in ten LMICs found that medicine utilization was often insufficient. Among the patients with coronary heart disease, 18.8% did not receive Aspirin, 51.9% did not use a β blocker, 60.2% did not obtain an ACE inhibitor and 79.2% did not receive a Statin (9). 

WHO’s Global Action Plan has set a goal to reach a 50% usage of the foregoing medicines by 2025, (10); Iran FDA, however, expects to increase the use of these medicines to 70% (11). So, these medicines should be widely available and affordable for all patients. There exist a multiple reasons why medicines are not used frequently: low availability and affordability, poor prescribing practices, and a lack of patient adherence (12). To reach the next step of the epidemiological transition in which deaths are prevented and the onset of disease is delayed, adequate treatment and preventive programs need to be implemented. In this regard, continuous pharmaceutical management along with combination therapy is fundamental. It has been shown that the recurrence of CVD could be diminished by 75% via adequate combination therapy (9). 

Global access has been defined as the continuous availability and affordability of medicines at public and private health facilities or medicine outlets within a one-hour walk from the patients’ houses. Affordability is the cost of a medication course pertaining to a specific disease relative to the daily wage of the lowest-paid unskilled government worker. Access to healthcare, an essential right of the patients, has been considered in international agreements and governmental policies (13). 

Several factors reduce access to medicines including high medication prices, poor availability, irrational medicine utilization, inequitable health financing, unreliable supply system, low medicine quality, and poor adherence on the part of the patients (14). 

Iran is located in the Middle East with an area of 1,648,000 square kilometers. According to WHO 2014 data, the total health expenditure in Iran was about 6.89%, for which the public sector accounts 2.84% and the private sector 4.05% of the gross domestic product (GDP). The amount of health expenditure per capita was approximately 1,082 US Dollars in 2014. In addition, the value for public health expenditure was nearly 41.2% of the total health expenditure and 17.53% of the governmental expenditure (15). 

The procurement system in Iran is similar between the public and private sectors and all pharmacies obtain their medicines from the same distributors in all sectors. Except for vaccines and orphan drugs, there is no centralized bulk purchasing or specific procurement system for public health facilities. The medicine prices are categorized in three levels (pharmacy, distributor, and importer/manufacturer) in the registration process and get updated once a year by the FDA in Iran. The patient treatment costs are identical in the public and private sectors with the FDA implementing national generic medicine policy to promote prescribing and dispensing of generic medicines parallel to permission for generic substitution by pharmacists. According to 2011 national population and housing census, the total population of Tehran province was 12,183,391 out of 75,149,669 (total population of Iran), meaning more than 16.21 percent of the total population are living in this province (16). Extensive studies have shown that through careful selection, such study sites can sufficiently represent the situation in the country as a whole (17). 

The aim of the present study, therefore, was to investigate the availability and affordability of cardiovascular medicines for secondary prevention in Tehran province.

## Methods

We conducted a survey on the availability and affordability of cardiovascular medicines for secondary prevention in Tehran province using a standardized methodology developed by WHO and Health Action International (HAI) (17, 18). 

The data on the availability and final medicine costs in public and private sector were collected from April to July of 2015. Atenolol 50 mg, Simvastatin 20 mg, and Captopril 25 mg were selected from the core list and Atorvastatin 20 mg, Furosemide 40 mg, Lisinopril 10 mg, and Nifedipine Retard 20 mg were chosen from the Eastern Mediterranean Regional office of the World Health organization (EMRO) regional lists for international comparison. The supplementary list was prepared on the basis of the local disease burden and local needs as determined by a community survey, while other factors, such as drug availability and utilization patterns, were further taken into account. For each medicine, we collected the data on the price and availability of the original brand (OB) and the lowest-price generic equivalent (LPG). 

A systematic sampling method was used to collect the data. Five geographical regions were selected in Tehran province: center, southwest, northwest, southeast, and northeast. In each area, the main government hospital and five public medicine outlets including both government hospital and outdoor public pharmacies were included. With regard to private pharmacies, we created a complete list of health facilities registered by the Ministry of Health and the Iranian Pharmacies Council. For each survey area, 18 pharmacies close to the public sectors were selected. If there were a number of private pharmacies close to each public facility, the pharmacies were selected randomly. An additional list of hospitals and pharmacies were maintained as a backup list. 

Data were collected by trained pharmacy students and supervised by the survey manager and the principal investigator. Data collectors were trained according to the WHO/HAI methodology. They visited medicine outlets and collected information on medicine availability and price through the use of a standard data collection form. The data collectors entered survey data into the already printed workbook provided as part of the WHO/HAI methodology. All the collected and completed forms containing data on medicine prices were checked on a daily basis.

We calculated the availability of the individual medicines as the percentage of sampled medicine outlets where the medicine was found. Data were reported in aggregate and by private versus public sector pharmacies. We also reported mean (average) availability for the list of medicines.

The availability of medicines and their prices were appraised within the public and the private sectors. Prior to data collection, an official letter was issued by the general director of Pharmaceutical and Narcotic Affair of Iran FDA for each trained pharmacists to ensure good cooperation between the staff and the team of researchers. For each medicine, data were collected based on the price and availability of the OB medicines and LPGs. In the public private sectors, only the retail prices were collected. Finally, the medicine prices were transformed into unit prices and recorded carefully on the day of data collection.

*Statistical analysis*

Upon finishing the field survey, a double input of data was inserted into the International Medicines Price Workbook software in order to reduce errors; the Workbook’s auto checker was further used in the verification process (17). 

The Workbook software calculated the median price ratio (MPR) for every medicine type in each sector. The Price criteria and MPR of each medicine was calculated via the Workbook developed by WHO/HAI only if the medicine was available in at least one facility. The MPR is the local median unit price of a medicine in comparison with the median unit price found in the Management Sciences for Health (MSH) Price Indicator Guide for the international reference price. The ideal value of MPR indicated acceptable local price ratios. Since MSH prices were not available for some supplementary medicines (Atorvastatin 40 mg, Enalapril 20 mg, Losartan 25 mg, and Propranolol 10 mg), Spanish manufacturers’ selling prices were employed as their reference prices (supplied by WHO/HAI project member Carmen Peres-Casas). Spain is known to have relatively low manufacturer selling prices (19-21). To describe the availability, the following ranges were used as reference (22):

< 30 %                    Very low

30 – 49 %               Low 

50 – 80 %               Fairly high

> 80 %                    High

*Assessment of affordability*

In accordance with WHO/HAI methodology, the affordability was assessed by calculating the number of days’ income required for patients to purchase any of the selected generic medicines for the CVD, namely 7-day treatment for an acute condition or 30-day treatment for a chronic condition. In general, treatments that cost a day’s income or less were considered affordable. Since the CVDs are considered chronic diseases, we calculated the cost of a 30-day treatment. 

The affordability was computed using the daily wage of the lowest-paid unskilled government worker, assessed as 237475 Rials per day (23) (USD 8.04) (24). 

## Results

One hundred and twenty samples were selected from the five survey areas including 10 outdoor public pharmacies, 20 public hospital pharmacies, and 90 private pharmacies (closest to each public healthcare facility).

**Table 1:** The availability of medicines in public hospital pharmacies, outdoor public pharmacies, and private pharmacies

	**Public Hospital Pharmacies**	**Outdoor Public Pharmacies**	**Private Pharmacies**
LPGs	85.2%	88.1%	87.8%
OBs	0.0%	0.0%	0.1%

**Table 2:** The availability of medicines of different lists

	**LPGs**			**OBs**		
	**Global list**	**Regional list**	**Supplementary list**	**Global list**	**Regional list**	**Supplementary list**
Public Hospital Pharmacies	97.7%	61.3%	89.6	0.0%	0.0%	0.0%
Outdoor public pharmacies	100%	70%	97.7%	0.0%	0.0%	0.0%
Private pharmacies	98.9%	69.4%	90.7%	0.0%	0.3%	0.1

**Table 3:** The availability of medicines among the five sectors in the province.

		**Public** ** Hospital Pharmacies **	**Outdoor Public pharmacies**	**Private pharmacies**
NW	LPGs	89.3	90.5	89.0
	OBs	0.0	0.0	0.0
SW	LPGs	79.8	88.1	86.9
	OBs	0.0	0.0	0.0
C	LPGs	86.9	85.7	87.6
	OBs	0.0	0.0	0.0
NE	LPGs	84.5	90.5	88.8
	OBs	0.0	0.0	0.5
SE	LPGs	85.7	85.7	82.1
	OBs	0.0	0.0	0.0

NW: Northwest, SW: Southwest, C: Center, NE: Northeast, SE: Southeast.

**Table 4:** MPRs of the surveyed medicines

	**MPRs of All Medicines**		**MPRs of Core Medicines**		**MPRs of Regional Medicines**		**MPRs of Supplementary Medicines**	
	**OB**	**LPG**	**OB**	**LPG**	**OB**	**LPG**	**OB**	**LPG**
Public Hospital Pharmacies		0.76		1.08		0.83		0.53
Outdoor Public Pharmacies		0.72		1.08		0.83		0.53
Private Pharmacies		0.72		1.08		0.83		0.53

**Table 5:** The affordability of the survey Medicines

**Medicines**	**No. of daily wage required to buy monthly requirement**
Aspirin 100 mg	≥ 0.1
Aspirin 80 mg	≥ 0.1
Atenolol 100 mg	0.1
Atenolol 50 mg	≥ 0.1
Atorvastatin 10 mg	0.2
Atorvastatin 20 mg	0.2
Atorvastatin 40 mg	0.2
Captopril 25 mg	0.1
Captopril 50 mg	0.1
Enalapril20 mg	0.1
Enalapril 5 mg	0.1
Furosemide 40 mg	0.1
Losartan 25 mg	0.1
Losartan 50 mg	0.1
Nifedipine retard 20 mg	0.1
Propranolol 10 mg	0.2
Propranolol 40 mg	0.1
Simvastatin20 mg	0.2

*Availability*

The mean availability was 85.2 in the public hospital pharmacies, 88.1 in outdoor public pharmacies, and 87.8 in the private pharmacies. The data also showed that the mean availability of OBs was 0.0% in public hospital pharmacies and outdoor pharmacies of public healthcare facilities and only 0.1% in private pharmacies. The OB of Atorvastatin was available in very few private pharmacies. 

Regardless of OB or generic medicine, the overall availability of the selected medicines was more than 80%. Table 1 demonstrates the availability of medicines in different sectors. 

As seen, the overall availability of medicines from the global list was 97.7%, 100%, and 98.9% in public hospital pharmacies, outdoor public pharmacies, and private pharmacies, respectively. With regard to the supplementary medicines intended for secondary prevention of CVD, the availability was 89.6%, 90.7%, and 90.7 %. The availability of the OB of global, regional, and supplementary lists was 0.0%, 0.3%, and 0.1% only in the private sector.

Regardless of innovator, generic, and branded generic, a high availability (>80%) of the medicines for CVD was seen in the survey sample.

*Percentage of medicine availability in the survey areas*

The availability of medicines in whole sectors of survey areas was further analyzed. All survey areas indicated availability of about 80%. There was only one survey area showing 0.5% availability of innovator brand of atorvastatin which was considered as very low available. In other survey areas, OBs were entirely unavailable except one area where LPG medicines were less than 80% available and others were more than 80% obtainable. Table 3 demonstrates the availability of different survey areas.

*Medicine prices*

The MPRs of all LPGs ranged from 0.53 to 1.08, indicating that the costs of LPGs approached the acceptable price line and were similar to the international reference prices. The MPRs for OB were not calculated due to the unavailability of the medicines. Furosemide 40 mg had the highest median prices of 3.29 times the IRP in all sectors. Losartan 25 mg had the lowest median price of 0.02, 0.03, and 0.03 times the IRP in the public hospital pharmacies, outdoor public pharmacies, and private sectors. Table 4 demonstrates the overview of the MRPs.

*Affordability*

The cost of purchasing LPGs in all surveyed was between 0.1 and 0.2 days’ wages, indicating that generic medicines are fairly accessible in Tehran province. The most affordable LPGs were Aspirin 80, 100 mg, and Atenolol 50 mg (which cost ≥ 0.1 days’ wages in all sectors). Considering the OB, the affordability was not calculated owing to the unavailability of the medicines in the studied sectors (Table 5).

## Discussion

The availability of cardiovascular medicines in both public and private sectors of Tehran province were studied. In Iran, the national generic medicine policy enforces prescribing and dispensing of generic medicines. Accordingly, generic medicine substitution by pharmacists is permitted. Furthermore, most of original brands of surveyed medicines are not registered by Iran FDA (25). 

In the present study, the generic availability did not differ between public and private pharmacies. The generic medicines had a high or fairly high availability in all sectors. 

In a previous study examining the availability, pricing, and affordability of cardiovascular medicines in 36 developing countries, the overall availability of five cardiovascular medicines was found to be low (average availability of 57.3% in the private sector and 26.3% in the public sector) (9).The results of our study, on the other hand, clearly demonstrated the high availability of CVD medicines in both private and public sectors.

The availability of the LPG medicines in the mentioned study and our survey was higher than OB products and these medicines were more obtainable in private sectors than in public sectors (9). Nevertheless, the availability among all sectors was almost the same.

Overall, the costs of cardiovascular medicines were high compared to international reference prices. With regard to LPGs, the average adjusted MPR for the entire group of medicines was 15.5 in the public sector and 30.2 in the private sector. Moreover, the median MRP for generic medicines was 0.72 in all sectors, demonstrating the lower price of the medicines in Tehran, in comparison to other countries. 

In another study examining the availability and affordability of CVD medicines (6), the availability of four CVD medicines in upper middle-income countries like Iran were 80% in urban and 73% in rural communities. Our results showed that in Iran, the availability of medicines were similar or even better than other upper middle-income countries.

A research explored the availability and affordability of chronic disease medications including one diuretic, two ACEs inhibitor and one Statin in six LMICs, showed that in public sectors, medicines were poorly obtainable and the majority of patients had to purchase the medicine from the private sector or forego the treatment if they were not able to afford. Although the availability of medicines was better in the private than in the public sector, still most countries need these medicines (12). In Iran, as demonstrated in the present research, medicines are more available in public and private sectors, comparisons to the studied countries.

However, OBs are not entirely available in public and private sectors which can be attributed to Iran’s health policy to augment the proportion of LPGs, decrease health expenditures, and improve drug affordability. The affordability data showed that a monthly prescription for all generic medicines was affordable to the lowest paid unskilled government worker in Tehran province. The OBs were not included in affordability calculation.

Studying the availability and affordability of cardiovascular medicines across 36 countries demonstrated that a month of chronic treatment of hypertension with one medicine costs 1.8 dayꞌs wages on average. In all countries, OB products and the medicines in private sectors were less affordable. Therefore cardiovascular medicines might be considered unaffordable in a significant proportion of countries (8). On the other hand, our results described that, in Iran, the generic medicines are fairly affordable for unskilled workers patients in both public and private sectors. 

Nevertheless, these results indicated the affordability of each single medicine, while a significant portion of the patients might require combination therapy to achieve treatment targets. Therefore, in one-income families, chronic treatment may become unaffordable if more than one family member needs chronic therapy (9). The affordability is likely to be overestimated as it does not take into account other medical costs, including professional fees, travel and time off work to visit the doctor. Our definition of affordability does not cover patient or household priorities. Even if these medicines are affordable, patients might still consider them unaffordable if they have other household expenditures they deem more important (e.g. treatment of other diseases, costs of housing, or education) (6). 

Of patients with known CVD across 18 countries in PURE study, only 686 (10%) use three of the recommended medicines while 205 (3%) use all four medicines. In LMICs, there is a strong link between the availability and affordability of these medicines and their usage. The utilization of the recommended medicines, however, is low in these countries even when the medicines are possibly available and affordable, reaching 18% in high-income countries (where these medicines are extensively available and affordable). Accordingly, though the availability and affordability of medicines are essential for their consumption, correcting these factors alone might not guarantee the optimum coverage of patients receiving all medicines (6, 7). 

In the WHO PREMISE study in MENA (Middle East, Northern Africa) region, Aspirin use was the most common form of prevention, prescribed to 81.3% of Iranians. Beta blockers were second, taken by 66%, in Iran. ACE-inhibitors were used by 27.9% and 28.1% employed Statins (5), corroborating the idea that even available and affordable medicines are not sufficient to prevent CVD.

WHO Global Action Plan has specified worldwide goals to achieve 80% availability and 50% usage of affordable essential medicines in non-communicable diseases by 2025 (26). The current rates of medicine utilization for secondary prevention fall substantially behind these goals. Overcoming such treatment gaps requires policies that make critical medicines available and affordable, and strategies to ameliorate their usage (e.g. enhancing access to healthcare providers, setting local targets for their use, and monitoring) (6). 

In Iran, physicians do not have positive attitudes toward the efficacy and safety of generic medicines, and more than 70% of them assume that the quality and efficacy of branded medicines are higher than those of generic ones. In addition, physicians may receive commissions and incentives from medical representatives of pharmaceutical companies to prescribe OBs (27). On the other hand, patients purchasing OBs from private outlets usually pay substantially more than they would pay for the generic equivalents. 

WHO has promoted the use of generic pharmaceutical products to reduce costs and improve access to healthcare. Nonetheless, the quality of pharmaceutical products available in the market in many developing countries varies, partly due to the paucity of clear and specific criteria for generic pharmaceuticals (28). Thus, continuous monitoring with standard and clear procedures is crucial to ensure the quality of the medicines. High quality generic medicines encourage patients and healthcare providers to use generic products instead of OBs.

The health system in Iran encounters many obstacles including insufficient investment and development in the pharmaceutical industry, unaffordability of medicines, and variation around the quality of domestic medicines, medicine shortage, counterfeit drugs, and unethical competition among pharmaceutical companies. To meet such challenges, the government should ensure that medicines correspond to the standards of quality, safety, and efficacy. The ministry of health should enforce strategies to enhance the quality and safety of generic medicines and provide evidence indicating the equivalency of generic and branded medicines in the market to promote the prescription and consumption of the former (29). 

## Conclusions

In Tehran province, the high availability of generic products in healthcare facilities reflects the success of the government’s policy to improve access to such medicines. National essential medicine lists and guidelines must be developed and implemented to maintain the availability and affordability of cardiovascular medicines. The availability of generic medication should be facilitated through educating healthcare professionals to follow standard treatment guidelines, encouraging generic prescription, increasing consumer awareness of the availability and affordability of the generics, implementing policies that make generic substitution compulsory, developing a remuneration policy for dispensing fees, and implementing fixed margins to support the utilization of generic products.

## Limitations

The present study was conducted at the time when Iranian population was still young. Since the CVD are age related, it has been supposed that Iran healthcare system will meet a big challenge over the next 20-30 years. A further obstacle was the paucity of essential drug lists and standard guidelines for management of CVD.
